# Cities with camera-equipped taxicabs experience reduced taxicab driver homicide rates: United States, 1996–2010

**DOI:** 10.1186/s40163-014-0004-3

**Published:** 2014-05-09

**Authors:** Cammie Chaumont Menéndez, Harlan Amandus, Parisa Damadi, Nan Wu, Srinivas Konda, Scott Hendricks

**Affiliations:** Analysis and Field Evaluations Branch, Division of Safety Research, National Institute for Occupational Safety and Health, Morgantown, West Virginia; University of Maryland, College Park, Maryland; Aspen of DC, Inc, Morgantown, West Virginia; National Institute for Occupational Safety and Health, AFEB, 1095 Willowdale Rd, MS 1811, Morgantown, West Virginia

**Keywords:** Taxicab driver homicides, Workplace violence, Public health, Robberies, Safety equipment, Intervention, Ecological study, Generalized estimating equations, Retrospective time series

## Abstract

**Background:**

Driving a taxicab remains one of the most dangerous occupations in the United States, with leading homicide rates. Although safety equipment designed to reduce robberies exists, it is not clear what effect it has on reducing taxicab driver homicides.

**Findings:**

Taxicab driver homicide crime reports for 1996 through 2010 were collected from 20 of the largest cities (>200,000) in the United States: 7 cities with cameras installed in cabs, 6 cities with partitions installed, and 7 cities with neither cameras nor partitions. Poisson regression modeling using generalized estimating equations provided city taxicab driver homicide rates while accounting for serial correlation and clustering of data within cities. Two separate models were constructed to compare (1) cities with cameras installed in taxicabs versus cities with neither cameras nor partitions and (2) cities with partitions installed in taxicabs versus cities with neither cameras nor partitions. Cities with cameras installed in cabs experienced a significant reduction in homicides after cameras were installed (adjRR = 0.11, CL 0.06-0.24) and compared to cities with neither cameras nor partitions (adjRR = 0.32, CL 0.15-0.67). Cities with partitions installed in taxicabs experienced a reduction in homicides (adjRR = 0.78, CL 0.41-1.47) compared to cities with neither cameras nor partitions, but it was not statistically significant.

**Conclusions:**

The findings suggest cameras installed in taxicabs are highly effective in reducing homicides among taxicab drivers. Although not statistically significant, the findings suggest partitions installed in taxicabs may be effective.

## Introduction

Taxicab drivers work in one of the most violent occupations in the United States (Richardson and Windau 2003). In 2010, the homicide rate for taxicab drivers was 7.4 per 100,000 workers, higher than the rate for police officers, 6.7 per 100,000 workers; the overall work-related homicide rate was 0.37 per 100,000 workers Bureau of Labor Statistics, (2010). Risk factors for homicides among taxicab drivers include working alone, working late hours, interacting with the public, and working with cash (NIOSH 1995; OSHA 2000). The taxicab industry and transportation regulators have relied heavily on the use of partitions or security cameras installed in taxicabs as effective deterrents against robbery. In the absence of city ordinances mandating the use of security cameras or partitions, some large companies require specific safety equipment as a company policy. Two reports have provided important, yet limited, insight into the potential effectiveness of security cameras and partitions in reducing taxicab driver robberies and assaults (Stone and Stevens 1999; Taxicab Advisory Group Committee 2004). Together, the reports provide summaries of decreased taxicab driver homicides and assaults across several cities equipping taxicabs with cameras ten years ago and findings on reduced assaults among taxicab drivers driving taxicabs equipped with partitions in one city 15 years ago. There remains scant scientific evidence supporting the effectiveness of either deterrent in reducing workplace violence outcomes among taxicab drivers.

The theoretical basis behind the installation of bullet-resistant partitions or security cameras is that decreasing the opportunity for the desired reward and/or increasing the probability of a perpetrator being caught would decrease robberies and assaults among taxicab drivers (Jeffrey 1971). Moreover, the tenets described in Crime Prevention Through Environmental Design (CPTED) and widely utilized for stationary business environments can be adapted for a mobile business environment (taxicabs) using the framework provided by Situational Crime Prevention which focuses on the opportunities and challenges for crime reduction in specific settings (Jeffrey 1971; Clarke 1992). In addition to the dearth of scientific evidence supporting safety equipment in taxicabs in reducing crime among taxicab drivers, the theoretical basis describing the effect of safety equipment as a crime deterrent for this population is often not described.

The primary objective of this study is to evaluate the effectiveness of security cameras in reducing taxicab driver homicides. The secondary objective is to evaluate the effectiveness of partitions in reducing taxicab driver homicides.

## Methods

### Study design

An ecological study design evaluated the impact of the two widely-used types of safety equipment designed to reduce robberies and homicides among taxicab drivers. A retrospective longitudinal time series analysis spanning 1996 through 2010 evaluated annual citywide taxicab driver homicide rates from 20 major cities representing the largest metropolitan areas. Using a population-based ranking of the metropolitan areas (Census 2000), industry regulators helped identify all camera and partition cities followed by eligible control cities.

### Data elements

The outcome, taxicab driver homicide rates, was aggregated at the city level and constructed from crime reports obtained from police departments (numerator) and the number of licensed taxicabs from municipal transportation regulators (denominator). A standardized search strategy tailored for municipal police crime departments was used to locate taxicab driver homicides using any one of the following criteria: (1) crime premise designated as ‘vehicle’, (2) name of licensed taxicab companies, (3) keywords ‘cab’, ‘taxi’ or ‘driver’ in the crime report or (4) news clippings reporting taxicab driver homicides. Each crime report provided by each municipal police department was reviewed for relevance by the first author. Information on installation year of camera or partition (or neither) was obtained directly from city transportation regulators. A city was designated annually as a “camera city” or “partition city” if >70% of the cabs had cameras or partitions during the study period; a cut point of 70% was decided *a priori* as it reflected the observed distribution across cities during early stages of study planning. Camera cities and partition cities were mutually exclusive as ordinances/company policies historically required either cameras or partitions (personal communication with industry regulators). Two covariates were included in the statistical analysis: the concurrent decline in homicide rates in the United States since 1990 (Hendricks et al. 2007) and the city-specific annual (background) homicide rates as provided by the Uniform Crime Reports (FBI 2010). These covariates were chosen as homicide rates among workers (in addition to taxicab drivers) were declining for reasons that were not necessarily associated with safety equipment use (Hendricks 2007) and homicide rates varied annually both within and across cities.

### Statistical analysis

Generalized estimating equations accounted for serial correlation and clustering of data within cities. Annual taxicab driver homicide rates were modeled on installation status (camera or partition) compared to control cities in two separate models adjusting for covariates. A separate model restricted to camera cities only compared post-installation versus pre-installation homicide rates. The Wald test statistic determined the significance of installation status. The natural logarithm of the number of licensed taxicabs by city per year was used as an offset variable. The taxicab driver homicide counts were assumed to follow a Poisson distribution; the offset variable provided the denominator used to calculate the homicide rates. The data were tested for dispersion and found to be slightly under-dispersed (scale = 0.92). Therefore, all confidence intervals were considered conservative in their range.

## Findings

### Taxicab driver homicides

Police crime report data were analyzed for 7 cities where cameras were installed in taxicabs, 6 cities where partitions were installed in taxicabs and 7 cities where neither partitions nor cameras were installed in taxicabs. From 1996 through 2010, 95 taxicab driver homicides were investigated by law enforcement authorities (Table 1). In camera cities there were 19 homicides pre-installation (occurring in 6 cities), and only seven homicides post-camera installation (occurring in 2 cities). The homicides occurring post-camera installation occurred in cities where one company had a policy requiring cameras rather than a citywide ordinance mandating camera installation in taxicabs. Six camera cities experienced a decrease in the taxicab driver homicide rate post-installation (the remaining camera city had no homicides during the entire study period).

**Table 1:** **Number of taxicab driver homicides, average number of licensed cabs and average taxicab driver homicide rate**

	**Number of Taxicab Driver Homicides**															**Average Number of Licensed Cabs**	**Average Taxicab Driver Homicide Rate** ^**§**^
	**1996**	**1997**	**1998**	**1999**	**2000**	**2001**	**2002**	**2003**	**2004**	**2005**	**2006**	**2007**	**2008**	**2009**	**2010**		
Cameras																	
City 1^¶^					1			1								382	0.35
City 2^¶^		1	1	1				1	1							1422	0.33
City 3		1	1			1			1						2	2092	0.19
City 4		1	1	1				1				1			1	1861	0.22
City 5^†^																669	0.00
City 6^¶^		1	1	1			1									1353	0.22
City 7^¶^		1					1		1							648	0.31
Partitions																	
City 8	3	2	1	1						1	2		N/A	N/A	N/A	1400	0.60
City 9		2														1685	0.09
City 10	2	5	1		2	2	2	2	1	1	1					6646	0.19
City 11				1					1	1						750	0.27
City 12				2	1	2	1	1	1		1					2169	0.28
City 13		2			1	1	1	3			1		1		1	1650	0.44
Control																	
City 14			1			1		1				1		1		1548	0.22
City 15				1					1						2	462	0.60
City 16			1				1		1							821	0.28
City 17^†^																250	0.00
City 18																332	0.00
City 19			2	1												915	0.23
City 20^†^	N/A	N/A	N/A													577	0.00

Shaded areas designate year of camera or partition implementation.

^§^per 1,000 cab drivers.

^¶^Cities with ordinance mandating taxicab cameras.

^†^These cities did not experience any taxicab driver homicides during the timespan studied.

N/A indicates crime reports not available for this time period.

### Statistical models of homicide rates

Modeling annual citywide taxicab driver homicide rates among camera cities post-installation compared with pre-installation revealed a statistically significantly reduced homicide rate (RR_unadj_ = 0.22; 95% CL 0.10, 0.52) (Table 2, Model 1). This reduction is more pronounced (RR_adj_ = 0.11; 95% CL 0.06, 0.24) after controlling for pre-existing annual changes in city taxicab driver homicide rates (“year”) and the background city homicide rate. A sub-analysis exploring changes in taxicab driver homicide rates among cities with only a company policy (rather than cities with an ordinance for which there were no homicides occurring post-installation) found a statistically significant reduction in rate (RR_unadj_ = 0.53; 95% CL 0.29, 0.99) that persisted (RR_adj_ = 0.40; 95% CL 0.13, 0.81) after covariate adjustment. Modeling annual citywide taxicab driver homicide rates on camera installation in camera cities compared with control cities post-installation versus pre-installation revealed a statistically significant reduction in homicide rate (RR_unadj_ = 0.31; 95% CL 0.14, 0.72) (Model 2) that persisted (RR_adj_ = 0.32; 95% CL 0.15, 0.67) after controlling for covariates. A sub-analysis exploring changes in taxicab driver homicide rates in cities where camera installation is not citywide but due to a company policy found a reduced rate compared to control cities in both unadjusted and adjusted models [(RR_unadj_ = 0.63; 95% CL 0.46, 0.88); (RR_adj_ = 0.58; 95% CL 0.38, 0.87)]. Modeling annual citywide taxicab driver homicide rates on partition installation in partition cities compared with control cities revealed a slightly higher rate (RR_unadj_ = 1.11; 95% CL 0.59, 2.09) which, after controlling for declining homicide rates and background crime rates, presented a reduction in taxicab driver homicide rate that was not statistically significant (RR _adj_ = 0.78; 95% CL 0.41, 1.47).

**Table 2:** **Statistical models describing intervention effects on city-wide taxicab driver homicide rates: United States, 1996—2010**

	Model 1*		***Model 2*** ^***†***^		***Model 3*** ^**§**^	
	**Unadjusted**	**Adjusted**	**Unadjusted**	**Adjusted**	**Unadjusted**	**Adjusted**
	**RR (95% CL)**	**RR (95% CL)**	**RR (95% CL)**	**RR (95% CL)**	**RR (95% CL)**	**RR (95% CL)**
*Variables*						
Cameras installed	0.22 (0.10, 0.52)	0.11 (0.06, 0.24)	0.31 (0.14, 0.72)	0.32 (0.15, 0.67)	—	—
Partitions installed	—	—	—	—	1.11 (0.59, 2.09)	0.78 (0.41, 1.47)
Year^¶^	—	1.09 (1.00, 1.19)	—	0.98 (0.92, 1.04)	—	0.88 (0.83, 0.94)
City homicide rate**	—	1.10 (1.01, 1.20)	—	1.12 (1.03, 1.23)	—	1.12 (1.06, 1.19)

*Rate ratio of taxicab driver homicide rates post-installation versus pre-installation of cameras.

^†^Rate ratio of taxicab driver homicide rates in camera cities compared to control cities.

^§^Rate ratio of taxicab driver homicide rates in partition cities compared to control cities.

^¶^The rate ratio represents an associated change in taxicab driver homicide rate for every increase of 1 year.

**The rate ratio represents an associated increase in taxicab driver homicide rate for every 1 unit increase in city homicide rate.

## Discussion

To our knowledge, this is the first study to conduct a large-scale evaluation of the effectiveness of a widely adopted type of taxicab safety equipment. Our findings suggest cities with camera-equipped taxicabs experience reduced rates of taxicab driver homicides post-installation and compared with cities that do not use cameras. Furthermore, those cities where camera-equipped taxicabs were mandated by ordinance experienced no taxicab driver homicides post-installation whereas the two cities that experienced taxicab driver homicides post-installation, although fewer, were in cities where the dominant (>70% of market) taxicab company implemented a policy of camera-equipped taxicabs. Findings suggest that cities with partition-equipped taxicabs may experience reduced taxicab driver homicide rates compared to nonpartition-equipped other cities.

The observance of a significant reduction in taxicab driver homicides in cities where taxicabs are equipped with cameras, and the more pronounced effect in cities where taxicabs are equipped with cameras as mandated by a city ordinance, was an encouraging one. Based on anecdotal evidence from transportation regulators in cities already mandating cameras by ordinance, transportation regulators are currently considering promulgating city ordinances for their cities that will mandate the use of security cameras in taxicabs. Our findings suggest cities where taxicabs are equipped with cameras could result in fewer taxicab driver homicides particularly in cities with an ordinance mandating taxicabs are equipped with cameras. Furthermore, these findings are consistent with a previous analysis examining news clippings to identify homicides (Chaumont Menéndez et al. 2013). Specifically, results of the news clippings analysis were concordant with the current study with respect to an observed effect of cameras in possibly reducing citywide taxicab driver homicide rates, but inconclusive for the effect of partitions.

Nearly all of the cities studied had installed partitions in taxicabs by 1996, which limited the data available to examine the pre-post differences in homicide rates. However, 15 years’ worth of homicide rates in partition cities compared to control cities, after adjusting for city homicide rates and temporal patterns in taxicab driver homicide rates, revealed no statistically significant difference.

The primary limitations to the study are due to the ecological study design: (1) we could not estimate individual risk of homicide for taxicab drivers across safety equipment types and (2) the lack of uniform data in the crime reports on homicide circumstances precluded more detailed analyses. Significant strengths of this study are the 15-year time span, systematic collection of data in 20 major US cities, pre-post design that includes use of comparison cities with neither camera-equipped nor partition-equipped taxicabs, and statistical analysis that accounts for serial correlation of data that adjusts for two crucial covariates. Additionally, in working with city regulators and counting only licensed drivers, we were able to assume the drivers were in taxicabs installed with required safety equipment. The study and its findings make a significant contribution to understanding the possible effect of taxicab safety equipment on taxicab driver homicides.

Taxicab driver personal safety in Seattle and King County, Final report and recommendations. The report of the Taxicab Advisory Group Committee on Driver Safety to the Director of the Department of Executive Administration for the city of Seattle. June 18, 2004.
